# Comparative analysis of basal metabolic rate measurement methods in overweight and obese individuals: A retrospective study

**DOI:** 10.1097/MD.0000000000039542

**Published:** 2024-08-30

**Authors:** Baris Karagun, Nuh Baklaci

**Affiliations:** a Department of Endocrinology and Metabolism Diseases, Faculty of Medicine, Baskent University, Adana, Turkey.

**Keywords:** basal metabolism, body composition, electrical impedance, obesity

## Abstract

The global prevalence of overweight and obesity is on the rise, presenting significant health challenges worldwide. Obesity is associated with various chronic diseases and imposes substantial economic burdens on society. Accurate assessment of basal metabolic rate (BMR) is crucial for effective weight management strategies. This retrospective study, conducted at Baskent University Hospital between October 2019 and October 2023, analyzed data from 133 overweight and obese individuals. Various methods including indirect calorimetry (IC), predictive equations (Harris–Benedict and Mifflin–St Jeor), and bioelectrical impedance analysis (BIA) were used to estimate BMR. Additionally, demographic, clinical, and body composition data were recorded. The mean BMR measured using IC was 1581 ± 322 kcal/day, which was significantly lower than the BMR estimated by other methods such as BIA (1765.8 ± 344.09 kcal/day), Harris–Benedict (1787.64 ± 341.4 kcal/day), and Mifflin–St Jeor equations (1690.08 ± 296.36 kcal/day) (*P* < .001). Among the predictive equations, the Mifflin–St Jeor method provided BMR estimates closest to the gold standard IC. When BMR measurement methods were compared to IC, 36.8% of measurements with the Harris–Benedict equation method, 50.4% with the Mifflin–St Jeor equation method, and 36.1% with the BIA method were within ± 10% agreement with IC measurements. Significant correlations were found between BMR and body composition parameters such as fat-free mass, muscle mass, and fat mass (*R* = 0.681, *P* < .001; *R* = 0.699, *P* < .001; *R* = 0.595, *P* < .001, respectively). Regression analysis identified that variables such as weight, height, body mass index, and muscle mass significantly predicted BMR measured by IC, accounting for 69.1% of the variance. This study underscores the challenges in assessing BMR in overweight and obese individuals. While IC remains the gold standard, predictive equations and BIA offer alternative methods. The Mifflin–St Jeor equation emerged as a practical option, closely aligning with IC results. However, discrepancies between methods and the influence of body composition highlight the importance of individualized approaches to BMR assessment in weight management strategies.

## 1. Introduction

The worldwide occurrence of overweight and obesity is steadily increasing, with over 2 billion individuals currently categorized as having excess body weight. This represents roughly 30% of the global population.^[1]^ Obesity and its associated disorders have emerged as significant health issues on a global scale.^[2]^ Obesity poses a significant health problem as it greatly raises the likelihood of developing diseases like type 2 diabetes mellitus, fatty liver disease, hypertension, coronary artery disease, dementia, osteoarthritis, obstructive sleep apnea, and various cancers. Consequently, it leads to a decrease in both quality of life and life expectancy.^[3]^ Obesity not only contributes to morbidity but also imposes significant economic burdens on society due to the chronic diseases associated with it.^[4]^ The primary contributors to obesity are excessive and unhealthy eating habits, coupled with physical inactivity.^[5]^ Dietary management is a crucial component of weight control, aiming to achieve a negative energy balance at a fundamental level.^[6]^ The total daily energy demands consist of 3 components: basal metabolic rate (BMR), energy expended for the thermic effect of meals, and energy due to physical activity.^[7]^ BMR represents the amount of energy needed on a daily basis to sustain fundamental biological activities.^[8]^ It represents the primary source of energy expenditure and serves as a crucial factor in estimating daily energy needs.^[9,10]^ A review of the literature suggests that accurately predicting BMR is crucial for determining energy requirements and designing effective weight management strategies, particularly in individuals with severe obesity.^[11–13]^ BMR fluctuates based on factors such as age, gender, weight, height, and is intricately linked to an individual’s body composition.^[14]^ The measurement of BMR can be conducted using either direct or indirect calorimetric techniques. Direct calorimetry directly quantifies heat output, whereas indirect calorimetry (IC) quantifies oxygen intake and carbon dioxide production, which are subsequently translated into their respective energy equivalents. Additionally, predictive equations can also be used to estimate BMR in adults.^[10]^

The efficacy of these techniques for assessing BMR has been evaluated across various patient cohorts.^[15,16]^ While IC being the gold standard, standardized equations like the Harris–Benedict and Mifflin equations offer convenient application but exhibit limited reliability.^[11,17]^ A systematic analysis of predictive equations in people with overweight and obesity revealed that the Harris–Benedict equation demonstrated the greatest accuracy for individuals with overweight and obesity, while the Lazzer equation showed the lowest bias for individuals with obesity (BMI > 30 kg/m^2^).^[18]^ In another systematic review, it was found that no single prediction equation provides accurate and precise resting energy expenditure estimates in all obese adults, and the review suggested using Mifflin equations for overweight and obese adults.^[19]^ The findings of a recent systematic review and meta-analysis, which synthesized results from multiple studies, demonstrated that the WHO and Harris & Benedict equations were the most accurate and precise in predicting BMR among individuals with severe obesity.^[20]^

Despite the studies in the literature, confusion still persists regarding the most accurate methods for measuring BMR across different populations. The aim of this study was to assess the consistency between BMR measured via IC and estimated BMR determined by bioelectrical impedance analysis (BIA), as well as through the Harris–Benedict and Mifflin–St Jeor equations. Furthermore, an investigation into the correlation between BMR measured using these methods and biochemical, demographic, and body composition factors was also conducted.

## 2. Methods

This retrospective study was conducted at Baskent University Hospital between October 2019 and October 2023. The study retrospectively examined the data of 133 individuals who were either obese or overweight. Patient demographics including age, gender, weight, height, body mass index (BMI), and lifestyle information were recorded. By dividing the weight (in kilograms) by the square of the height (in meters), BMI was calculated. The hemogram and blood biochemistry parameters were assessed at 8 am. The following parameters were included in the evaluation: fasting plasma glucose, alanine aminotransferase, and creatinine. The study was approved by the Institutional Ethics Committee of Baskent University and it was carried out in compliance with the ethical principles outlined in the Declaration of Helsinki (KA24/171).

### 2.1. Bioelectrical impedance analysis (BIA)

A Tanita (BC-420MA, Tanita Corp., Tokyo, Japan) brand BIA device was used to assess the body composition of the patients. This device was utilized to measure patients’ body fat percentage, fat-free mass, fat mass, muscle mass, body water content, and metabolic rate. Measurements were taken via electrodes placed on the soles of the patients’ feet after they removed their shoes and socks.

### 2.2. Indirect calorimetry (IC)

In this study, basal metabolic rate (BMR) was measured using IC (The Fitmate™, Cosmed, Rome, Italy) under controlled conditions to ensure accurate and reliable results. Participants were required to fast for at least 12 hours prior to the measurement to eliminate the thermic effect of food. Measurements were conducted in a thermoneutral environment, ensuring a stable temperature to avoid any influence on metabolic rate. Participants were asked to rest in a supine position for at least 30 minutes before the measurement to ensure they were in a true basal state. The calorimetry device measured oxygen consumption (VO_2_) and carbon dioxide production (VCO_2_) over a period of 20 to 30 minutes. By adhering to these stringent conditions, we ensured that the BMR values obtained accurately reflected the participants’ BMRs, devoid of external influences such as recent physical activity or dietary intake.

### 2.3. Harris–Benedict equation

BMR was estimated using the Harris–Benedict equation,^[21]^ which takes into account age, gender, weight, and height of the participants. The equation used for men was:


$$\begin{array}{llll} BMRmen= & \;88.362+\left(13.397\times weight\;in\;kg\right)\; & +\left(4.799\times height\;in\;cm\right)\; & -(5.677\times age\;in\;years) \end{array}$$

For women, the equation used was:


$$\begin{array}{llll} BMRwomen= & \;447.593+\left(9.247\times weight\;in\;kg\right)\; & +\left(3.098\times height\;in\;cm\right)\; & -(4.330\times age\;in\;years) \end{array}$$

### 2.4. Mifflin–St Jeor equation

BMR was estimated using the Mifflin–St Jeor equation,^[22]^ which takes into account age, gender, weight, and height of the participants. The equations used for men and women are as follows:

For men:


$$\begin{array}{lll} BMRmen= & \;\left(10\times weight\;in\;kg\right)+\left(6.25\times height\;in\;cm\right)\; & -\left(5\times age\;in\;years\right)+5 \end{array}$$

For women:


$$\begin{array}{lll} BMRwomen= & \;\left(10\times weight\;in\;kg\right)+\left(6.25\times height\;in\;cm\right)\; & -\left(5\times age\;in\;years\right)-161 \end{array}$$

### 2.5. Statistical analysis

The data were analyzed using the SPSS (Statistical Package for Social Sciences) 22.0 package program. In descriptive analyses, frequency data were presented as number (n) and percentage (%), while numerical data were presented as mean ± standard deviation (minimum–maximum). The normal distribution of numerical data was assessed through histogram and Q–Q plot graphs in the sample size and data distribution. The distribution of normally distributed numerical data in dependent 2 groups was examined using the Paired samples *T* test. The degree of agreement between the gold standard and other BMR measurement methods was evaluated using the Bland–Altman plot. The agreement limits were defined as mean ± 2 SD. The relationship between normally distributed 2 numerical variables was examined using Pearson correlation analysis. Univariate and backward multivariate linear regression analysis were conducted to assess the predictive properties of independent predictors on BMR parameters. Correlation relationships were classified as follows: *R* = 0.05–0.30 for low or weak correlation, *R* = 0.30–0.40 for low-moderate correlation, *R* = 0.40–0.60 for moderate correlation, *R* = 0.60–0.70 for strong correlation, *R* = 0.70–0.75 for very strong correlation, and *R* = 0.75–1.00 for perfect correlation. A statistical significance level of *P* < .05 was considered for all tests.^[23]^

## 3. Results

The study included 133 overweight and obese participants, with 78.2% (n = 104) being female. The average age was 42.26 ± 12.13 years (ranging from 18–80 years), and the average BMI was 35.54 ± 5.57 kg/m² (ranging from 25.1–47.00 kg/m²). The mean BMR assessed using IC was 1581.00 ± 322.00 kcal/day (range: 658–2705 kcal/day). BMR measured with BIA was higher, averaging 1765.80 ± 344.09 kcal/day (range: 1219–2812 kcal/day). The fat percentage measured by BIA averaged 41.66 ± 6.80%, with fat-free mass at 56.59 ± 11.28 kg, muscle mass at 53.77 ± 10.64 kg, and bone mass at 3.07 ± 2.74 kg (Table 1).

**Table 1:** The demographic and clinical characteristics of patients.

Variables	Results (n = 133)
Gender (female); n(%)	104 (78.2)
Age (years)	42.26 ± 12.13 (18–80)
Fasting blood glucose (mg/dL)	94.89 ± 12.61 (75–148)
ALT (IU/L)	26.21 ± 15.24 (8–94)
Creatinine (mg/dL)	0.72 ± 0.14 (0.42–1.69)
Height (cm)	166.20 ± 8.83 (150–188)
Weight (kg)	98.65 ± 19.74 (66–153)
BMI (kg/m^2^)	35.54 ± 5.57 (25.1–47)
IC BMR (kcal)	1581 ± 322 (658–2705)
Harris–Benedict BMR (kcal)	1787.64 ± 341.4 (1212.6–2881.8)
Mifflin–St Jeor BMR (kcal)	1690.08 ± 296.36 (1066.5–2526.4)
BIA BMR (kcal)	1765.80 ± 344.09 (1219–2812)
Fat percentage (%)	41.66 ± 6.8 (20.8–54.9)
Fat free mass (kg)	56.59 ± 11.28 (31.2–88.1)
Muscle mass (kg)	53.77 ± 10.64 (39.2–83.8)
Fat mass (kg)	41.77 ± 12.59 (10.9–75)
Bone mass (kg)	3.07 ± 2.74 (2.1–34)

Mean ± SD (min–max).

BIA = bioelectrical impedance analysis, BMI = body mass index, BMR = basal metabolic rate, IC = indirect calorimetry.

**Figure 1.**
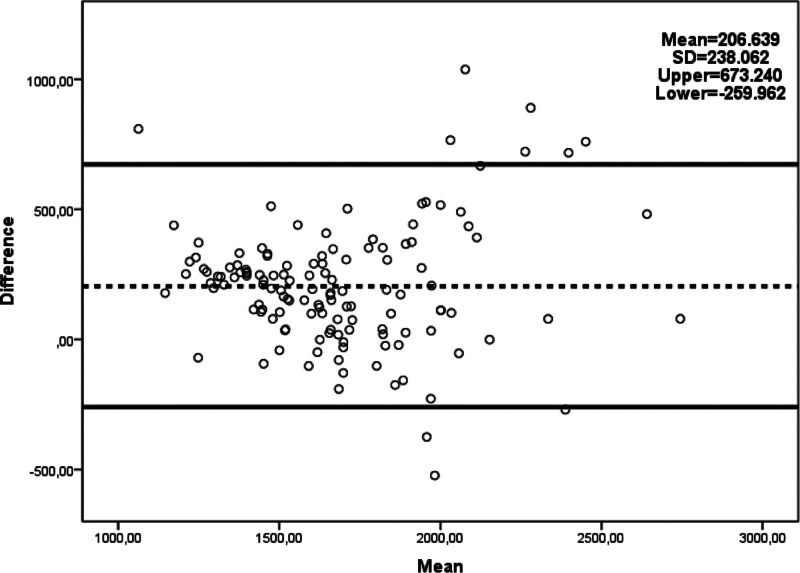
Bland–Altman plot of agreement between predicted BMR by the Harris–Benedict equation and BMR result by IC.

**Figure 2.**
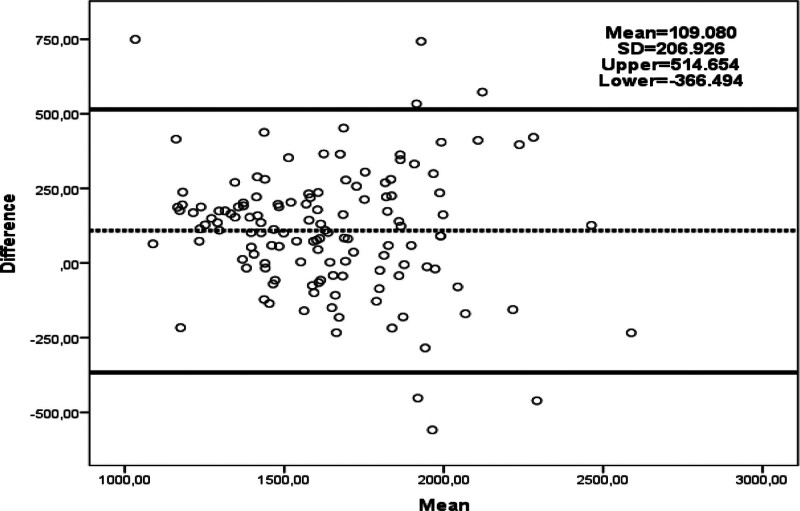
Bland–Altman plot of agreement between predicted BMR by the Mifflin–St Jeor equation and BMR result by IC.

**Figure 3.**
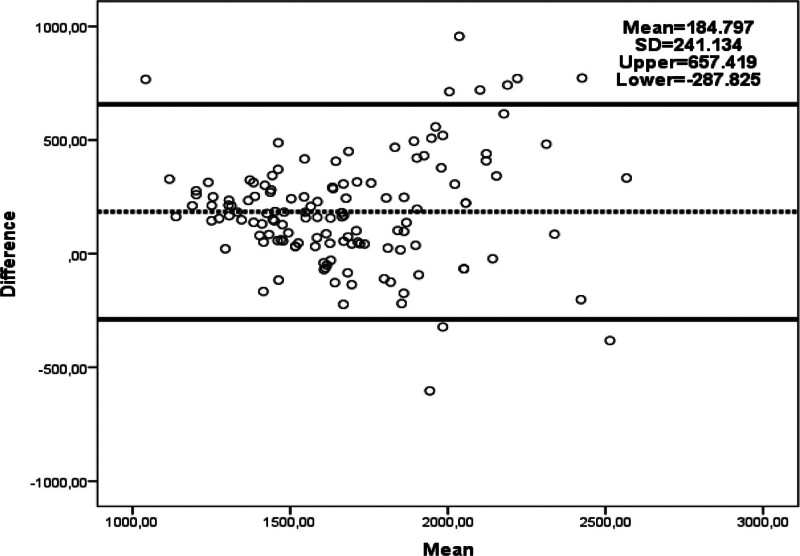
Bland–Altman plot of agreement between predicted BMR by BIA and BMR result by IC.

Comparing BMR measurement methods (Table 2), IC yielded significantly lower BMR values than other methods (*P* < .001). The Harris–Benedict method calculated higher BMR values than both the Mifflin–St Jeor and BIA methods (*P* < .001 and *P* = .008, respectively). BIA also provided higher BMR values compared to Mifflin–St Jeor (*P* < .001). Figures 1, 2, and 3 illustrate the agreement results between BMR measured with IC and other methods. When evaluated against the gold standard IC, 36.8% of Harris–Benedict, 50.4% of Mifflin–St Jeor, and 36.1% of BIA measurements were within ± 10% of IC measurements (Table 3).

**Table 2:** Comparison of BMR measurement methods.

	Mean ± SD	Correlation	*P* *
IC BMR (kcal/day) Harris–Benedict BMR (kcal/day)	1581 ± 322 1787.64 ± 341.401	0.744	**<.001**
IC BMR (kcal/day) Mifflin–St Jeor BMR (kcal/day)	1581 ± 322 1690.08 ± 296.36	0.779	**<.001**
IC BMR (kcal/day) BIA BMR (kcal/day)	1581 ± 322 1765 ± 344.09	0.740	**<.001**
Harris–Benedict BMR (kcal/day) Mifflin–St Jeor BMR (kcal/day)	1787.64 ± 341.401 1690.08 ± 296.36	0.983	**<.001**
Harris–Benedict BMR (kcal/day) BIA BMR (kcal/day)	1787.64 ± 341.401 1765 ± 344.09	0.963	**.008**
Mifflin–St Jeor BMR (kcal/day) BIA BMR (kcal/day)	1690.08 ± 296.36 1765 ± 344.09	0.957	**<.001**

Bold *P* values indicate statistical significance.

Mean** ± **SD (min–max).

BIA = bioelectrical impedance analysis, BMR = basal metabolic rate, IC = indirect calorimetry.

* Paired samples *T* test.

**Table 3:** Evaluation of measurement methods according to IC.

	Lower than IC Measurement; n (%)	±%10 IC; n (%)	Higher than IC measurement; n (%)
Harris–Benedict BMR	23 (17.3)	49 (36.8)	110 (82.7)
Mifflin–St Jeor BMR	36 (27.1)	67 (50.4)	97 (72.9)
BIA BMR	23 (17.3)	48 (36.1)	110 (82.7)

BIA = bioelectrical impedance analysis, BMR = basal metabolic rate, IC = indirect calorimetry.

Table 4 presents the relationships between BMR assessment methods and other variables. Significant positive correlations were found between BMR measured with IC and fat-free mass (*R* = 0.681, *P* < .001), muscle mass (*R* = 0.699, *P* < .001), and fat mass (*R* = 0.595, *P* < .001). Similar significant positive correlations were observed for Harris–Benedict, Mifflin–St Jeor, and BIA methods with various body composition metrics.

**Table 4:** Relationship between BMR measurement methods and biochemical and clinical data.

		IC BMR (kcal)	Harris–Benedict BMR (kcal)	Mifflin–St Jeor BMR (kcal)	BIA BMR (kcal)
Age (years)	*r* *P*	−0.128 .143	−0.332 **<.001**	−0.350 **<.001**	−0.242 **.005**
Fasting blood glucose (mg/dL)	*r* *P*	0.196 **.024**	0.089 .308	0.088 .313	0.146 .093
ALT (IU/L)	*r* *P*	0.179 **.040**	0.272 **.002**	0.241 **.005**	0.290 **.001**
Creatinine (mg/dL)	*r* *P*	0.129 .137	0.290 **.001**	0.266 **.002**	0.356 **<.001**
Height (cm)	*r* *P*	0.631 **<.001**	0.761 **<.001**	0.793 **<.001**	0.787 **<.001**
Weight (kg)	*r* *P*	0.791 **<.001**	0.891 **<.001**	0.923 **<.001**	0.866 **<.001**
BMI (kg/m^2^)	*r* *P*	0.560 **<.001**	0.585 **<.001**	0.616 **<.001**	**0.549** **<.001**
Fat percentage (%)	*r* *P*	0.237 **.006**	0.054 .0541	0.132 .131	−0.064 .466
Fat free mass (kg)	*r* *P*	0.681 **<.001**	0.911 **<.001**	0.895 **<.001**	0.964 **<.001**
Muscle mass (kg)	*r* *P*	0.699 **<.001**	0.921 **<.001**	0.909 **<.001**	0.984 **<.001**
Fat mass (kg)	*r* *P*	0.595 **<.001**	0.574 **<.001**	0.637 **<.001**	0.485 **<.001**
Bone mass (kg)	*r* *P*	0.172 **.047**	0.250 **.004**	0.246 **.004**	0.301 **<.001**

*r* = Pearson correlation coefficient.

Bold *P* values indicate statistical significance.

BIA = bioelectrical impedance analysis, BMI = body mass index, BMR = basal metabolic rate, IC = indirect calorimetry.

One-way regression analysis showed that age, height, weight, BMI, fat percentage, fat-free mass, muscle mass, fat mass, and bone mass were predictive factors of BMR (*P* < .005) (Table 5). The backward multivariate regression analysis revealed that the model encompassing all variables had an explanatory power of 69.1%, indicating these data as significant predictive factors of BMR (Table 6).

**Table 5:** Univariate regression analysis results for IC BMR.

	B (% 95 CI)	Beta	t	*P*	Zero-order
Age	−3.387 (−7.937 to 1.162)	−0.128	−1.473	.143	−0.128
Height (cm)	22.996 (18.109–27.883)	0.631	9.308	**<.001**	0.631
Weight (kg)	12.905 (11.181–14.629)	0.791	14.809	**<.001**	0.791
BMI (kg/m^2^)	32.34 (24.068–40.162)	0.560	7.734	**<.001**	0.560
Fat percentage (%)	11.222 (3.274–19.170)	0.237	2.793	**.006**	0.237
Fat free mass (kg)	19.445 (15.835–23.055)	0.681	10.656	**<.001**	0.681
Muscle mass (kg)	21.13 (17.389–24.871)	0.699	11.172	**<.001**	0.699
Fat mass (kg)	15.216 (11.666–18.767)	0.595	8.478	**<.001**	0.595
Bone mass (kg)	20.186 (0.244–40.127)	0.172	2.002	**.047**	0.172

B: unstandardized coefficient, Beta: standardized coefficient.

Bold *P* values indicate statistical significance.

BMI = body mass index, BMR = basal metabolic rate.

**Table 6:** Multivariate backward regression analysis results for IC BMR.

Model	R	R square	Adjusted R Square	Std. error of the estimate	R square change	F change	df1	df2	Sig. F change	Durbin Watson
**1**	0.831	0.691	0.668	185.513	0.691	30.522	9	123	<0.001	
**2**	0.831	0.691	0.671	184.768	<0.001	0.007	1	123	0.935	
**3**	0.831	0.691	0.673	184.087	<0.001	0.080	1	124	0.777	
**4**	0.830	0.690	0.675	183.623	0.001	0.365	1	125	0.547	
**5**	0.830	0.689	0.676	183.174	0.001	0.380	1	126	0.539	
**6**	0.829	0.687	0.678	182.835	0.001	0.527	1	127	0.469	2.017

1. Predictors: (constant), bone mass (kg), fat mass (kg), age (years), height (cm), fat-free mass (kg), fat percentage (%), BMI (kg/m^2^), muscle mass (kg), weight (kg).

2. Predictors: (constant), bone mass (kg), fat mass (kg), age (years), height (cm), fat percentage (%), BMI (kg/m^2^), muscle mass (kg), weight (kg).

3. Predictors: (constant), bone mass (kg), fat mass (kg), age (years), height (cm), fat percentage (%), BMI (kg/m^2^), weight (kg).

4. Predictors: (constant), bone mass (kg), fat mass (kg), age (years), fat percentage (%), BMI (kg/m^2^), weight (kg).

5. Predictors: (constant), fat mass (kg), age (years), fat percentage (%), BMI (kg/m^2^), weight (kg).

6. Predictors: (constant), fat mass (kg), fat percentage (%), BMI (kg/m^2^), weight (kg).

BMI = body mass index; BMR = basal metabolic rate.

## 4. Discussion

The aim of this study is to compare different methodologies for estimating BMR with respect to biochemical parameters, body composition, and the gold standard IC method. The findings of this study shed light on the challenges and complexities of assessing BMR in overweight and obese individuals. The remarkable finding of the study was that alternative methods yielded higher BMR values in comparison to IC, recognized as the gold standard method. Among the methodologies discussed in the study, the Mifflin–St Jeor Equation method yielded the most closely aligned BMR estimation with IC.

As the prevalence of overweight and obesity continues to rise globally, it becomes increasingly important to understand the factors influencing BMR measurement in order to develop effective weight management strategies.^[24,25]^ While IC is considered the gold standard, predictive equations and BIA can be used to estimate BMR.^[24,26,27]^ IC is considered the gold standard for BMR measurement, boasting high validity. However, its widespread adoption is hindered by factors such as limited availability, high cost, time requirements, and the need for specialized personnel. As a result, weight loss interventions typically rely on prediction equations to estimate BMR.^[11,28,29]^ The literature extensively evaluates the adequacy and reliability of these methods.^[11,12,29,30]^ Luy et al found that in obese Filipinos with type 2 DM or prediabetes, both the Harris–Benedict equation and BIA have a tendency to overestimate BMR when compared to measurements acquired using IC.^[30]^ In our study, we observed results consistent with those reported in the study by Luy et al We found that BMR measured using predictive equations (Harris–Benedict and Mifflin equations) and BIA yielded significantly different results. Specifically, BMR measured with IC was consistently lower compared to estimates from predictive equations and BIA. This highlights the limitations of relying solely on predictive equations or BIA for assessing BMR, especially in overweight and obese populations. The discrepancies observed between BMR measurement methods underscore the importance of using a multimodal approach to assess energy expenditure in overweight and obese individuals. Because overestimating BMR could potentially hinder the success of weight loss programs, particularly among obese individuals. Literature indicates that these equations tend to overestimate BMR across various patient groups.^[31]^ In our study, we observed that the Mifflin–St Jeor method provided the BMR calculation closest to that obtained via IC. A systematic review revealed that the Mifflin–St Jeor equation exhibits a greater likelihood of predicting resting metabolic rate within 10% of the measured value when compared to other equations assessed. According to this systematic review, it is advised to use the Mifflin–St Jeor equation if a prediction formula is used.^[11]^ The results regarding the Mifflin–St Jeor equation in our study were in line with the findings reported in this systematic review.

BMR is primarily influenced by body composition, specifically fat-free mass and fat mass, as well as factors such as gender, age, physical activity, and nutritional status. Fat-free mass is the primary factor that determines BMR.^[14]^ Molnar et al find that fat-free mass primarily influences resting metabolic rate, although age, gender, and fat mass also significantly contribute to it.^[32]^ Similarly, Johnstone et al also found that factors contributing to the variability in BMR include fat-free mass, fat mass, age, and circulating thyroxine.^[33]^ In our study, we observed a noteworthy correlation between fat-free mass, muscle mass, fat mass, and the calculation methods of BMR. Univariate and multivariate regression analyses revealed that age, height, weight, BMI, fat percentage, fat-free mass, muscle mass, fat mass, and bone mass were predictive factors of BMR. These findings underscore the influence of body composition on BMR and highlight the importance of considering individual characteristics when estimating energy expenditure.

This study has several limitations that should be considered when interpreting the results. First of all, the retrospective design introduces inherent biases and makes it challenging to establish causal relationships between variables. The sample size might not encompass the full diversity within the overweight and obese population, potentially impacting the robustness of the findings. The study solely included overweight and obese participants. The results may not be applicable to individuals with normal weight or underweight conditions. Finally, while body composition variables were included in the analysis, other factors influencing body composition, such as hormonal status and lifestyle factors, were not fully accounted for.

In conclusion, our study highlights the challenges and complexities associated with assessing BMR in overweight and obese individuals. Although IC is considered the gold standard, alternative methods such as predictive equations and BIA offer alternative methods for estimating BMR. The Mifflin–St Jeor equation is notable for its results being closest to the IC and its practical usability. However, discrepancies between measurement methods and the influence of body composition underscore the need for a comprehensive approach to BMR assessment. By considering individual characteristics, clinicians can better tailor weight management strategies to meet the needs of overweight and obese individuals. Further research is warranted to explore additional factors that may influence BMR in this population and to validate the findings of this study in larger and more diverse cohorts.

## Acknowledgments

We would like to express our deepest gratitude to “Prof Dr Okan Sefa Bakiner’’ for his invaluable guidance and encouragement throughout the development of this study.

## Author contributions

**Conceptualization:** Baris Karagun.

**Data curation:** Baris Karagun.

**Formal analysis:** Baris Karagun.

**Investigation:** Baris Karagun.

**Methodology:** Baris Karagun.

**Resources:** Baris Karagun.

**Supervision:** Nuh Baklaci.

**Validation:** Baris Karagun.

**Visualization:** Baris Karagun, Nuh Baklaci.

**Writing – original draft:** Baris Karagun.

**Writing – review & editing:** Baris Karagun, Nuh Baklaci.

## References

[1] Caballero B. Humans against obesity: who will win? Adv Nutr. 2019;10:S4–9. 30721956 10.1093/advances/nmy055 PMC6363526

[2] Seidell JC Halberstadt J. The Global burden of obesity and the challenges of prevention. Ann Nutr Metab. 2015;66(Suppl. 2):7–12. 10.1159/000375143 26045323

[3] Blüher M. Obesity: global epidemiology and pathogenesis. Nat Rev Endocrinol. 2019;15:288–98. 30814686 10.1038/s41574-019-0176-8

[4] Tremmel M Gerdtham UG Nilsson PM Saha S. Economic burden of obesity: a systematic literature review. Int J Environ Res Public Health. 2017;14:435. 28422077 10.3390/ijerph14040435 PMC5409636

[5] Haslam DW James WPT. Obesity. Lancet (London, England). 2005;366:1197–209. 16198769 10.1016/S0140-6736(05)67483-1

[6] Chao AM Quigley KM Wadden TA. Dietary interventions for obesity: clinical and mechanistic findings. J Clin Invest. 2021;131:e140065. 33393504 10.1172/JCI140065 PMC7773341

[7] Levine JA. Measurement of energy expenditure. Public Health Nutr. 2005;8:1123–32. 16277824 10.1079/phn2005800

[8] Ng JCM Schooling CM. Effect of basal metabolic rate on lifespan: a sex-specific Mendelian randomization study. Sci Rep. 2023;13:7761. 37173352 10.1038/s41598-023-34410-6 PMC10182013

[9] Ferro-Luzzi A. The conceptual framework for estimating food energy requirement. Public Health Nutr. 2005;8:940–52. 16277813 10.1079/phn2005789

[10] Shetty P. Energy requirements of adults. Public Health Nutr. 2005;8:994–1009. 16277816 10.1079/phn2005792

[11] Frankenfield D Roth-Yousey L Compher C. Comparison of predictive equations for resting metabolic rate in healthy nonobese and obese adults: a systematic review. J Am Diet Assoc. 2005;105:775–89. 15883556 10.1016/j.jada.2005.02.005

[12] Sabounchi NS Rahmandad H Ammerman A. Best-fitting prediction equations for basal metabolic rate: informing obesity interventions in diverse populations. Int J Obes (Lond). 2013;37:1364–70. 23318720 10.1038/ijo.2012.218 PMC4278349

[13] Horgan GW Stubbs J. Predicting basal metabolic rate in the obese is difficult. Eur J Clin Nutr. 2003;57:335–40. 12571669 10.1038/sj.ejcn.1601542

[14] Lazzer S Bedogni G Lafortuna CL. Relationship between basal metabolic rate, gender, age, and body composition in 8,780 White Obese Subjects. Obesity (Silver Spring, Md.). 2010;18:71–8. 19478787 10.1038/oby.2009.162

[15] Flancbaum L Choban PS Sambucco S Verducci J Burge JC. Comparison of indirect calorimetry, the Fick method, and prediction equations in estimating the energy requirements of critically ill patients. Am J Clin Nutr. 1999;69:461–6. 10075331 10.1093/ajcn/69.3.461

[16] Boullata J Williams J Cottrell F Hudson L Compher C. Accurate determination of energy needs in hospitalized patients. J Am Diet Assoc. 2007;107:393–401. 17324656 10.1016/j.jada.2006.12.014

[17] Tignanelli CJ Andrews AG Sieloff KM. Are predictive energy expenditure equations in ventilated surgery patients accurate? J Intensive Care Med. 2019;34:426–31. 28382850 10.1177/0885066617702077

[18] Macena M de L Paula DT da C da Silva Júnior AE. Estimates of resting energy expenditure and total energy expenditure using predictive equations in adults with overweight and obesity: a systematic review with meta-analysis. Nutr Rev. 2022;80:2113–35. 35551409 10.1093/nutrit/nuac031

[19] Madden AM Mulrooney HM Shah S. Estimation of energy expenditure using prediction equations in overweight and obese adults: a systematic review. J Human Nutr Dietetics. 2016;29:458–76. 10.1111/jhn.12355 26923904

[20] Campos TA de M Mariz VG Mulder AP Curioni CC Bezerra FF. Adequacy of basal metabolic rate prediction equations in individuals with severe obesity: a systematic review and meta-analysis. Obes Rev. 2024;25:e13739. 38548479 10.1111/obr.13739

[21] Roza AM Shizgal HM. The Harris Benedict equation reevaluated: resting energy requirements and the body cell mass. Am J Clin Nutr. 1984;40:168–82. 6741850 10.1093/ajcn/40.1.168

[22] Mifflin MD St Jeor ST Hill LA Scott BJ Daugherty SA Koh YO. A new predictive equation for resting energy expenditure in healthy individuals. Am J Clin Nutr. 1990;51:241–7. 2305711 10.1093/ajcn/51.2.241

[23] Hayran M. Basic Statistics for Health Research. 1st ed. Ankara: Offset Printing. Art Ofset; 2011.

[24] Henry CJK. Basal metabolic rate studies in humans: measurement and development of new equations. Public Health Nutr. 2005;8:1133–52. 16277825 10.1079/phn2005801

[25] Hill JO Wyatt HR Peters JC. The importance of energy balance. Eur Endocrinol. 2013;9:111–5. 29922364 10.17925/EE.2013.09.02.111 PMC6003580

[26] Delsoglio M Achamrah N Berger MM Pichard C. Indirect calorimetry in clinical practice. J Clin Med. 2019;8:1387. 31491883 10.3390/jcm8091387 PMC6780066

[27] Radley D Cooke CB Fuller NJ. Validity of foot-to-foot bio-electrical impedance analysis body composition estimates in overweight and obese children. Int J Body Compos Res. 2009;7:15–20. 20396615 PMC2854815

[28] Haugen HA Chan LN Li F. Indirect calorimetry: a practical guide for clinicians. Nutr Clin Pract. 2007;22:377–88. 17644692 10.1177/0115426507022004377

[29] Frankenfield DC Rowe WA Smith JS Cooney RN. Validation of several established equations for resting metabolic rate in obese and nonobese people. J Am Diet Assoc. 2003;103:1152–9. 12963943 10.1016/s0002-8223(03)00982-9

[30] Luy SC Dampil OA. Comparison of the Harris-Benedict Equation, bioelectrical impedance analysis, and indirect calorimetry for measurement of basal metabolic rate among adult obese filipino patients with prediabetes or type 2 diabetes mellitus. J ASEAN Fed Endocr Soc. 2018;33:152–9. 33442121 10.15605/jafes.033.02.07 PMC7784146

[31] BonganhaVLibardiCASantosCF. Predictive equations overestimate the resting metabolic rate in postmenopausal women. J Nutr Health Aging. 2013;17:211–4.23459971 10.1007/s12603-012-0395-3

[32] Molnár D Schutz Y. The effect of obesity, age, puberty and gender on resting metabolic rate in children and adolescents. Eur J Pediatr. 1997;156:376–81. 9177980 10.1007/s004310050618

[33] Johnstone AM Murison SD Duncan JS Rance KA Speakman JR. Factors influencing variation in basal metabolic rate include fat-free mass, fat mass, age, and circulating thyroxine but not sex, circulating leptin, or triiodothyronine. Am J Clin Nutr. 2005;82:941–8. 16280423 10.1093/ajcn/82.5.941

